# Cytological and molecular characterization of wheat lines carrying leaf rust and stem rust resistance genes *Lr24* and *Sr24*

**DOI:** 10.1038/s41598-024-63835-w

**Published:** 2024-06-04

**Authors:** Jianbo Li, Haixia Guan, Yuqi Wang, Chongmei Dong, Richard Trethowan, Robert A. McIntosh, Peng Zhang

**Affiliations:** 1 Plant Breeding Institute, School of Life and Environment Sciences, The University of Sydney, 107 Cobbitty Road, Cobbitty, NSW 2570 Australia; 2 Triticeae Research Institute, Sichuan Agricultural University, Chengdu, 611130 Sichuan China

**Keywords:** Genetics, Plant sciences

## Abstract

Previous studies showed that Australian wheat cultivars Janz and Sunco carry leaf rust and stem rust resistance genes *Lr24* and *Sr24* derived from *Thinopyrum ponticum* chromosome arm 3AgL. However, the size of the alien segments carrying *Lr24* and *Sr24* in the lines were not determined. In this study, we used non-denaturing fluorescence in situ hybridization (ND-FISH), genomic in situ hybridization (GISH), and PCR-based landmark unique gene (PLUG) markers to visualize the alien segments in Janz and Sunco, and further compared them with the segments in US cultivars Agent and Amigo. The fraction length (FL) of the alien translocation in Agent was 0.70–1.00, whereas those in Janz, Sunco, and Amigo were smaller, at FL 0.85–1.00. It was deduced that the alien gene *R*^*Ag*^ encoding for red grain color and rust resistance genes *Lr24* and *Sr24* on chromosome arm 3AgL were in bins of FL 0.70–0.85 and 0.85–1.00, respectively. We retrieved and extracted nucleotide-binding site-leucine-rich repeat (NBS-LRR) receptor genes corresponding to the region of *Lr24* and *Sr24* on chromosomes 3E, and 3J, 3J^s^ and 3St from the reference genome sequences of *Th. elongatum* and *Th. intermedium*, respectively. A set of molecular markers developed for *Lr24* and *Sr24* from those extracted NBS-LRR genes will provide valuable information for fine mapping and cloning of these genes.

## Introduction

The hard red winter wheat cultivar Agent, CI 13523, carrying a spontaneous wheat-*Thinopyrum ponticum* translocation T3DS.3DL-3AgL was released by Oklahoma Agricultural Experiment Station in 1967^1^. At that time, Agent was resistant to all known races of *Puccinia triticina* (*Pt*), the leaf rust pathogen, and moderately resistant to “some races of stem rust” caused by *P. graminis* f. sp. *tritici* (*Pgt*)^1^. The leaf rust and stem rust resistances in Agent were derived from *Th. ponticum*, shown to be completely linked, and designated *Lr24* and *Sr24*, respectively^2^. The translocation carrying resistance was incorporated into wheat cultivars, and the Agent source of *Lr24* and *Sr24* was widely exploited in the Americas and South Africa. However, attempts to use the resistance in Australia failed because all derivatives had red grain color.

Sears^3^ used induced homoeologous recombination to produce several 3D-3Ag translocation lines carrying *Lr24* in Chinese Spring (CS) which has the red grain allele *R1* on chromosome 3D. Thus, the source of the red grain allele (i.e. *R1* or *R*^*Ag*^) in these lines was unknown^4^. Following discussions with Sears, four of these lines were backcrossed with white seeded Australian genotypes and white seeded rust resistant lines were obtained when Sears’ lines CS 3D-3Ag#3 and CS 3D-3Ag#14 were used as donors^5^. Based on chromosome pairing studies Sears^3^ had predicted that these lines had the smallest 3Ag chromosome segments. The first white-seeded Australian cultivar carrying *Lr24* and *Sr24*, Torres, was released in Queensland in 1983. Subsequently, more than 60 cultivars, including Janz and Sunco, carrying *Lr24* and *Sr24* were released and widely grown throughout Australia. This is one of the most successful examples of overcoming linkage drag in exploiting high value resistance genes^4,6^. *Lr24* remained effective in Australia for almost 20 years until a virulent pathotype was detected in 2000^7^. *Sr24* continues to be effective and remains an important source of stem rust resistance despite its early failure against race Ug99 in Eastern Africa and earlier failure in South Africa in 1987^8^.

*Sr24* and *Lr24* were identified in some white seeded, stem rust resistant Australian backcross lines with Amigo as donor. Amigo was believed to carry resistance from cereal rye; however, some derivatives also had leaf rust resistance that was later attributed to *Lr24*. There was no evidence of an association with red grain color. Jiang et al.^9^ and Friebe et al.^10^ reported that Amigo had a non-homoeologous translocation T1BL.1BS-3AgL with the 3Ag segment lacking the *R*^*Ag*^ allele. In this study, we used ND-FISH, GISH, PLUG markers, and comparative genomic analysis to: (1) visualize the alien segments carrying *Lr24* and *Sr24* in Agent, Janz, Sunco and Amigo; (2) further map the *Lr24*/*Sr24* and *R*^*Ag*^ genes to smaller regions on chromosome arm 3AgL; and (3) develop a set of new chromosome 3AgL-specific NBS-LRR-related molecular markers within the same bin as the *Lr24*/*Sr24* genes.

## Results

### Cytogenetic identification of four wheat lines carrying* Lr24* and *Sr24*

**Figure 1:**
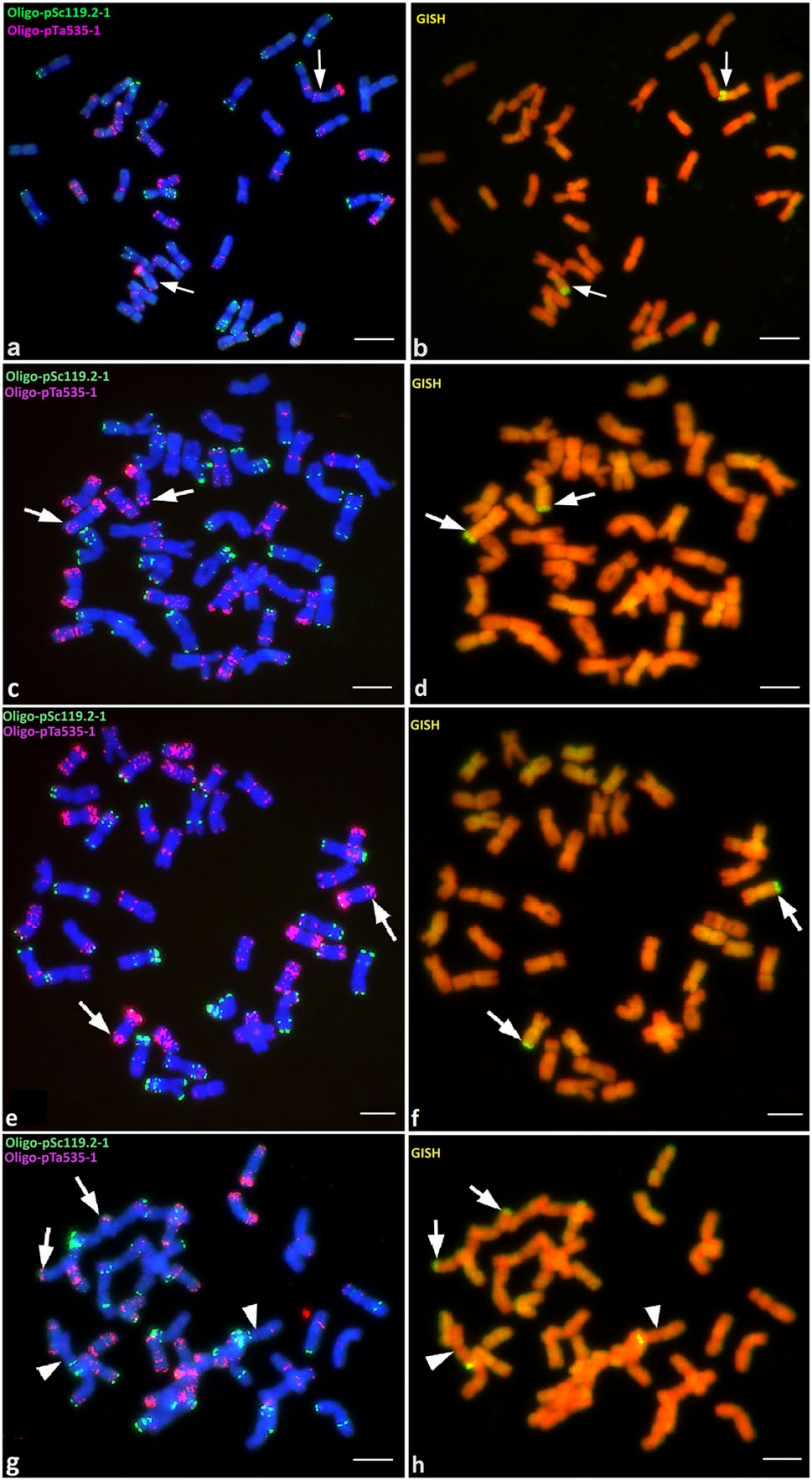
FISH-GISH images of metaphase chromosomes of four wheat lines carrying *Lr24* and *Sr24*, Agent (**a,b**), Janz (**c,d**), Sunco (**e,f**) and Amigo (**g,h**). Probes Oligo-pSc119.2-1 (green) and Oligo-pTa535-1 (red) were used in FISH (**a,c,e,g**) and *Pseudoroegneria stipifolia* (St) genomic DNA was used as the probe (yellow-green) for GISH (**b,d,f,h**). (**a–f**) Arrows point to the breakpoint in T3DS.3DL-3AgL. (**g,h**) Arrows point to the breakpoint in T1BL.1BS-3AgL, and arrowheads point to the breakpoint for T1RS.1AL. Bars, 10 μm.

ND-FISH and GISH images of mitotic metaphase cells of Agent, Janz, Sunco and Amigo are shown in Fig. 1. ND-FISH analysis using Oligo-pSc119.2-1 and Oligo-pTa535-1 allowed us to identify individual chromosomes. Agent (Fig. 1a), Janz (Fig. 1c), and Sunco (Fig. 1e) all had 42 chromosomes. Combined with GISH analysis using *Pseudoroegneria stipifolia* (St) genomic DNA as a probe (Fig. 1b,d,f), it was revealed that Agent, Janz and Sunco each had 40 wheat chromosomes and two wheat-*Thinopyrum* translocation chromosomes, T3DS.3DL-3AgL. The 3AgL chromosome segments in Janz and Sunco were similar in size, and smaller than that in Agent. As expected, Amigo had 38 wheat chromosomes, two T1BL.1BS-3AgL wheat-*Thinopyrum* translocation chromosomes and a pair of T1RS.1AL wheat-cereal rye translocation chromosomes (Fig. 1g,h). The 3AgL segment in Amigo was similar in size as those in Janz and Sunco.

### Locating the *Lr24*/*Sr24* and red grain color genes on chromosome arm 3AgL

**Figure 2:**
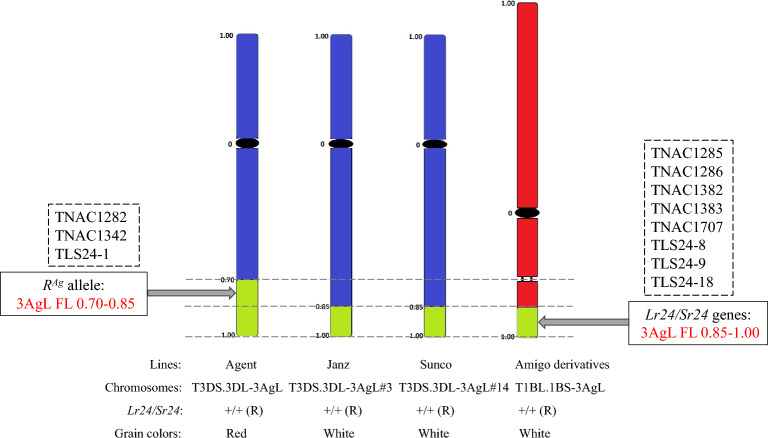
Chromosomal locations of the red grain color gene *R*^*Ag*^ and *Lr24/Sr24* genes in chromosome arm 3AgL. Dashed lines separate chromosome 3AgL-specific molecular markers specific for *Lr24*/*Sr24* and *R*^*Ag*^, respectively. Green, blue, and red colors indicate *Th. ponticum* chromosome 3AgL segments, and wheat chromosomes 3D and 1B, respectively. +**/+ **(R) indicates that the line carries *Lr24* and *Sr24*. Red and white, grain colors.

The chromosomal segment sizes and breakpoint positions of four translocation lines carrying *Lr24* and *Sr24* were determined using GISH. As shown in Table 1 and Fig. 2, the 3Ag segment in translocation chromosome T3DS.3DL-3AgL in Agent was in the long arm bin FL 0.70–1.00, and that in Janz, Sunco and Amigo were in bins FL 0.85–1.00. Because Agent, Janz, Sunco, and Amigo all carry *Lr24* and *Sr24*, they were mapped to 3AgL bin FL 0.85–1.00 (Fig. 2). In addition, Agent had red grain color, whereas Janz, Sunco and Amigo derivatives had white grain color, so the gene encoding the red grain color in Agent (*R*^*Ag*^) must be in 3AgL bin FL 0.70–0.85 (Fig. 2).

**Table 1:** Analysis of 3AgL chromosomal segment sizes and breakpoint positions in four wheat lines carrying *Lr24/Sr24.*

Line	Translocation type	3AgL segment size	FL
Agent	T3DS.3DL-3AgL	(0.70–1.00)3AgL	(0.30 ± 0.008)L
Janz	T3DS.3DL-3AgL#3	(0.85–1.00)3AgL	(0.15 ± 0.005)L
Sunco	T3DS.3DL-3AgL#14	(0.85–1.00)3AgL	(0.15 ± 0.005)L
Amigo	T1BL.1BS-3AgL	(0.85–1.00)3AgL	(0.15 ± 0.004)L

The position of the centromere was considered 0, and the terminal end of long arm of T3DS.3DL-3AgL in Agent was considered 1.00. L and S represent long and short arm, respectively. The column “FL” shows the fraction length. ± Standard error.

### Screening of chromosome 3AgL-specific PLUG markers identifying *R*^*Ag*^, *Lr24 *and *Sr24*

**Figure 3:**
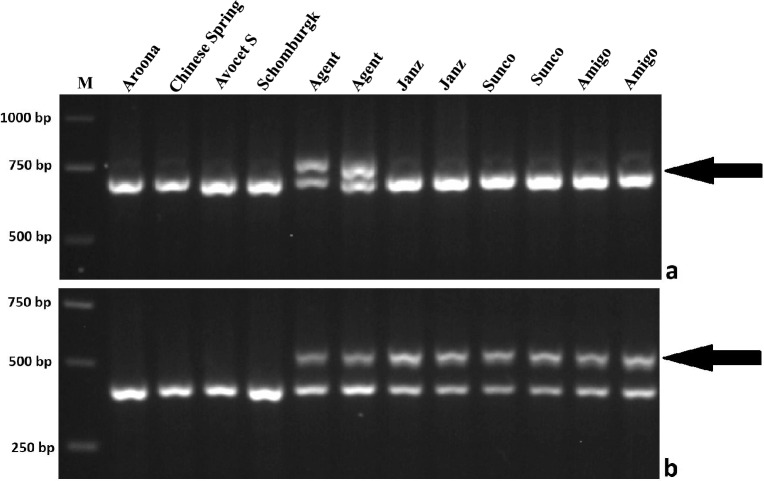
PCR amplification patterns of two representative chromosome 3AgL-specific markers in common wheat and *Lr24/Sr24*-carrying lines. (**a**) TNAC1282 (*Taq* I); (**b**) TNAC1707 (*Taq* I). M, GeneRuler (Thermo Scientific, Waltham, MA) 1 kb DNA Ladder. Arrows point to chromosome arm 3AgL-specific bands. Uncropped gel images are provided in Supplementary Information (Supplementary Fig. 1).

Twenty-four PLUG primer pairs from the long arms of homoeologous group 3 chromosomes were used to identify chromosome 3AgL-specific markers. Five markers, *TNAC1285*, *TNAC1286*, *TNAC1382*, *TNAC1383*, and *TNAC1707*, generating specific fragments in Agent, Janz, Sunco and Amigo, but not in Aroona, CS, Avocet S (AvS) and Schomburgk were chromosome 3AgL-specific and were in 3AgL bin FL 0.85–1.00 (Table 2). Another two markers, *TNAC1282* and *TNAC1342*, amplified specific bands in Agent but not in Janz, Sunco and Amigo, or Aroona, CS, AvS and Schomburgk, indicating their locations in 3AgL bin FL 0.70–0.85 along with *R*^*Ag*^ (Table 2). The images for two representative PLUG markers, *TNAC1282* and *TNAC1707*, are shown in Fig. 3. The primer sequences of the chromosome 3AgL-specifc PLUG markers are listed in Table 2.

**Table 2:** Sequences of seven pairs of *Thinopyrum ponticum* chromosome arm 3AgL-specific PLUG primers.

Primer name	Primer sequence (5ʹ–3ʹ)	3AgL bin (FL)	Physical location (bp)			Restriction enzyme	Product size (bp)	Annealing temperature (°C)
			Ta_3A	Ta_3B	Ta_3D			
TNAC1282	F: TGCCTTTCCACAGACGATTT	0.70–0.85	682,973,431–682,974,139	723,029,284–723,030,101	546,246,215–546,246,920	*Taq*I	750	60
	R: TAGAGTCGAGCCTCTGGAATG							
TNAC1342	F: CGAGCTCGATGTTGAAGATGT	0.70–0.85	708,325,338–708,325,434	763,773,520–763,773,616	573,691,047–573,691,143	*Taq*I	800	60
	R: CTGGTGGTCAGCAACAAAGAC							
TNAC1707	F: ATCCGAGGAAACAGTTCCATT	0.85–1.00	716,367,330–716,367,734	776,358,350–776,358,754	581,728,116–581,728,136	*Taq*I	500	60
	R: TACTCGGCTCTGGTCTTTGG							
TNAC1285	F: CTAATATTTGCCGGGCTTCTT	0.85–1.00	727,841,272–727,841,292	821,143,069–821,144,064	600,174,937–600,175,912	*Hae*III	800	60
	R: CCGTGGTCCAGGCCTACC							
TNAC1286	F: TCCGGTGTTTGAAGAAACTTG	0.85–1.00	730,677,052–730,678,524	826,367,931–826,369,170	603,686,640–603,686,660	*Hae*III	400	60
	R: GTCCGGGAGGAGATCCAG							
TNAC1382	F: CCTGAAGGCTGTGAGATGCTA	0.85–1.00	745,404,364–745,404,364	841,965,430–841,965,447	613,811,639–613,811,656	*Hae*III	490	60
	R: ACCGATGGCACCACCAAG							
TNAC1383	F: GCGGTCGATCTTCTTCAAGTC	0.85–1.00	752,794,520–752,795,561	851,512,028–851,513,189	619,065,943–619,067,283	*Hae*III	500	60
	R: TCAGATGGACTATGGGAGCAC

### Development of chromosome 3AgL-specific NBS-LRR-related markers

**Figure 4:**
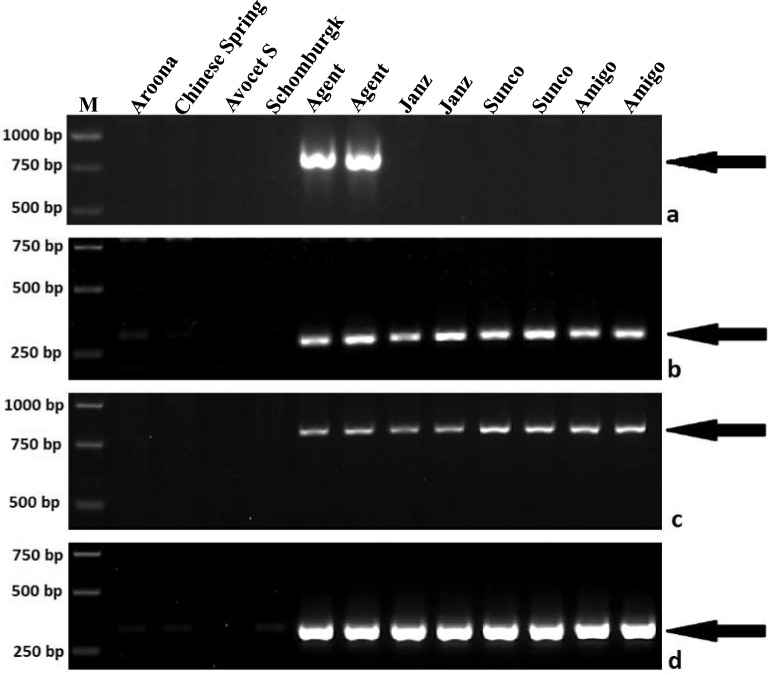
PCR amplification patterns of the newly developed chromosome arm 3AgL-specific NBS-LRR-related markers. (**a**) TLS24-1; (**b**) TLS24-8; (**c**) TLS24-9; (**d**) TLS24-18. M, GeneRuler (Thermo Scientific, Waltham, MA) 1 kb DNA Ladder. Arrows point to chromosome arm 3AgL-specific bands. Uncropped gel images are provided in Supplementary Information (Supplementary Fig. 2).

As showed in Table 2, the 3AgL bin FL 0.85–1.00 containing *Lr24* and *Sr24* contained five chromosome 3AgL-specific PLUG markers. Based on the syntenic relationships among *Th. elongatum*, *Th. intermedium* and *Th. ponticum* species, the 3AgL bin FL 0.85–1.00 carrying *Lr24* and *Sr24* were located into the physical intervals 597.72–675.27, 586.76–664.51, 464.48–526.13 and 435.61–493.34 Mb in chromosomes 3E, 3J, 3J^s^ and 3St, respectively. We then retrieved and extracted 26, 30, 42 and 30 NBS-LRR genes (related to disease response) in the respective physical intervals (Table S1). Using Primer-BLAST software we designed and developed four pairs of chromosome 3AgL-specific primers from the CDS sequences of three NBS-LRR genes (Table 3). Three of these markers mapped to 3AgL bin FL 0.85–1.00, and one mapped to 3AgL bin FL 0.70–0.85. PCR amplification results for these markers are shown in Fig. 4. Combining all results, eight markers were in bin FL 0.85–1.00 containing *Lr24* and *Sr24*, and three were in bin FL 0.70–0.85 containing *R*^*Ag*^ (Fig. 2).

**Table 3:** Primer sequences of newly developed chromosome arm 3AgL-specific NBS-LRR-related makers.

Primer name	Primer sequences (5ʹ–3ʹ)	3AgL bin (FL)	Corresponding gene		Product size (bp)	Annealing temperature (°C)
			Gene ID	Gene annotated function		
TLS24-1F	GTTGAACCATCCACTCAAGGTG	0.70–0.85	*Tel3E01G811400*	NBS-LRR-related	800	60
TLS24-1R	CTTCTGTAAGTCCGCGACCAATG					
TLS24-8F	TGTACGGCACTGCAGAAACT	0.85–1.00	*Thint.J03G585000.1*	NBS-LRR-related	300	63
TLS24-8R	AACAGATCCATGGAGGACGAG					
TLS24-9F	AGACCGCCGTCTGCAATTAT	0.85–1.00	*Thint.J03G585000.1*	NBS-LRR-related	800	62
TLS24-9R	AAACAGATCCATGGAGGACG					
TLS24-18F	AAGGTTTCGTTAGCAAAGCACG	0.85–1.00	*Thint.V03G517700.1*	NBS-LRR-related	350	62
TLS24-18R	ATTCGTGGAGCCTCTCCAGT

## Discussion

Although neither *Lr24* nor *Sr24* is a source of durable rust resistance, they are still useful for resistance breeding in many parts of the world, especially in combination with other genes. Several publications reported the development of molecular markers linked to *Lr24* and/or *Sr24*^11–14^. These resistance genes originated from *Th. ponticum* mainly in the form of chromosome T3DS.3DL-3AgL translocations^10,15^. Janz and Sunco are white-seeded Australian wheat cultivars with CS 3D-3Ag#3 and CS 3D-3Ag#14 as donors of *Lr24* and *Sr24*. Although Sears^3^ predicted that CS 3D-3Ag#3 and CS 3D-3Ag#14 might carry smaller alien segments than that in Agent based on chromosome pairing studies, their exact chromosomal structures were not determined. Zwart et al.^16^ constructed a genetic linkage map of chromosome 3D using 111 doubled haploid lines derived from a cross between synthetic hexaploid wheat accession CPI133872 and Janz. Their finding that *Lr24* was flanked by *P37M54e* and *Xgwm169b* at 0.4 cM and 1.0 cM, respectively, inspired our curiosity to characterize the 3AgL chromosomal segment size in Janz and Sunco. In the present study, ND-FISH and GISH revealed that different translocation events in Janz (Fig. 1c,d) and Sunco (Fig. 1e,f) were of similar size and significantly smaller than the translocation in Agent (Fig. 1a,b), as predicted by Sears^3^. Based on the cytological results, *R*^*Ag*^ and *Lr24* and *Sr24* were in 3AgL bins FL 0.70–0.85 and 0.85–1.00, respectively (Fig. 2). Combined with the PLUG marker results, the 3AgL bin FL 0.85–1.00 containing *Lr24* and *Sr24* was estimated to be at least 60 Mb (Table S1). Due to the presence of the *Ph1* (pairing homoeologous 1) gene in wheat, it is highly unlikely to have recombination between the *Th. ponticum* segment in the translocation chromosome T3DS.3DL-3AgL and homoeologous wheat chromosome regions in Janz and Sunco, as also mentioned by others^2,11,12,14^. All these results made us question the high recombination frequency between the translocated 3AgL chromosomal fragment carrying *Lr24* in Janz and homoeologous wheat chromosome 3D reported by Zwart et al.^16^.

PLUG markers^17^ based on orthologous gene conservation between rice and wheat have proven to be highly efficient and reliable molecular markers for confirming homoeologous relationships between wheat and alien chromosomes^18–20^. In this study, we used 24 PLUG primer pairs identified at the distal ends of the long arms of homoeologous group 3 chromosomes to genotype Agent, Janz, Sunco and Amigo, and found that five markers amplified *Thinopyrum*-specific bands. Two additional PLUG markers amplified fragments specific to Agent (Fig. 3, Table 2), indicating that: (1) the translocated *Thinopyrum* chromosomal segments in Agent, Janz, Sunco and Amigo belonged to homeologous group 3 (3AgL); and (2) the 3AgL chromosomal segments in Janz, Sunco and Amigo were similar in size, but smaller than that in Agent.

With the rapid development of sequencing technologies and availability of whole genome sequences of *T. aestivum*, *Hordeum vulgare* L. and *Secale cereale*, comparative genomic analysis is becoming increasingly achievable in developing molecular markers for use in non-sequenced species or in fine mapping of target genes^21–24^. In this study, based on the syntenic relationships among *Th. elongatum* chromosome 3EL, *Th. intermedium* chromosomes 3JL, 3J^s^L and 3StL, and the current *Th. ponticum* chromosome 3AgL, we developed four pairs of chromosome 3AgL-specific NBS-LRR-related primers (Fig. 4, Table S1) from the coding sequences (CDS) of three NBS-LRR genes in the *Lr24/Sr24* region. Moreover, the eight *Th. ponticum* chromosome 3AgL-specifc molecular (five PLUG and three NBS-LRR) markers developed in this study will be useful in future fine mapping and cloning *Lr24* and/or *Sr24*. No study thus far has separated the two genes indicating relatively close linkage, but we have no reason to believe they are the same gene.

Grain color is an important trait affecting flour yield and quality in wheat. Previous studies indicated that red-seeded wheats are more tolerant to preharvest sprouting than white-seeded wheats^25^. Hence Agent or another of Sears’ translocation lines may be preferred as donors of *Sr24* and *Lr24* in locations where red seeded wheat is preferred. In countries such as Australia and India where white seededness is considered a key aspect of quality or marketing advantage the use of these resistance genes was made possible only by their separation from *R*^*Ag*^. In hindsight, mutation of *R*^*Ag*^ in an appropriate derivative lacking *R2* and *R3* from Agent or in other Sears’ derivatives other than CS 3D-3Ag#3 and CS 3D-3Ag#14 could have achieved the same result.

## Methods

### Plant materials

The materials used in this study included four common wheat (*Triticum aestivum* L., AABBDD, 2*n* = 6*x* = 42) cultivars Aroona, Chinese Spring, Avocet S and Schomburgk, and four cultivars carrying *Lr24* and *Sr24*, namely Agent, Janz, Sunco and Amigo. Agent is a hard red winter wheat cultivar carrying a spontaneous wheat-*Th. ponticum* translocation T3DS.3DL-3AgL^1^. Janz and Sunco are white-seeded Australian wheat cultivars with CS 3D-3Ag#3 and CS 3D-3Ag#14 translocations, respectively, as donors of *Sr24* and *Lr24*. The red-seeded wheat cultivar Amigo with a T1AL.1RS Robertsonian translocation also carried a 3Ag chromosome segment, but as a T1BL.1BS-3AgL translocation^9^. The above materials are maintained at the Plant Breeding Institute, The University of Sydney.

### Cytological analysis

#### Chromosome preparation

Slides for examination of mitotic metaphase chromosomes of Agent, Janz, Sunco and Amigo were prepared according to the procedure in Lang et al.^26^ with minor modifications.

#### Non-denaturing fluorescence in situ hybridization (ND-FISH)

ND-FISH with oligonucleotide probes Oligo-pSc119.2-1 and Oligo-pTa535-1 was used to identify individual wheat chromosomes^27^. Oligo-pSc119.2-1 and Oligo-pTa535-1 were labelled with 6-carboxyfluorescein (6-FAM) generating green signals and 6-carboxytetramethylrhodamine (Tamra) generating red signals, respectively. Chromosomes were counterstained with 4′,6-diamidino-2-phenylindole (DAPI) (Invitrogen Life Science, Carlsbad, CA, USA) in Vectashield (Vector Laboratories, Burlingame, CA) and pseudo-colored blue. Slides were analyzed with a Zeiss Axio Imager epifluorescence microscope. Images were captured with a Retiga EXi CCD (charge-coupled device) camera (QImaging, Surrey, BC, Canada) operated with Image-Pro Plus version 7.0 software (Media Cybernetics Inc., Bethesda, MD, USA) and processed with Photoshop version CC 2022 software (Adobe Systems, San Jose, CA).

#### Genomic in situ hybridization (GISH)

Sequential GISH was performed after stripping off the oligo probes in 50% FA/1 × SSC and 50% FA/0.5 × SSC for 10 min each at 42 °C. GISH followed the procedure of Zhang et al.^28^. Total genomic DNA of *Ps. stipifolia* (StSt, 2*n* = 2*x* = 14, PI 314058, The National Small Grains Collection, USDA-ARS; kindly provided by Dr. L. Qi, USDA-ARS, ND) was used as probe, which was labelled with biotin-16-dUTP (Roche Diagnostics Australia, Castle Hill, NSW, Australia) using nick translation. Unlabeled total genomic DNA of CS wheat was used as blocker with a probe:blocker ratio of 1:120. Signals were detected with fluorescein–avidin DN (Vector Laboratories, Burlingame, CA). Chromosomes were counterstained with DAPI and pseudo-colored red.

### Alien segment size and breakpoints in translocation lines

To estimate the size and breakpoints of the *Th. ponticum* 3AgL chromosomal segments in lines carrying *Lr24* and *Sr24*, 10 cells at mitotic metaphase from each translocation line were photographed. Arm lengths were measured using ImageJ v2.0.0 software^29^, and the ratio between the length of 3AgL chromosomal segments and the long arm of translocation chromosome T3DS.3DL-3AgL in Agent was calculated as described by Endo and Gill^30^. The position of the centromere was considered zero, and the terminal end of long arm of T3DS.3DL-3AgL in Agent was considered one. Standard error calculations for fraction length (FL) measurements were performed with the software Excel 2021 (IBM Corporation, New York).

### PLUG marker analysis

Genomic DNA was extracted from young leaves of Aroona, CS, AvS, Schomburgk, Agent, Janz, Sunco, and Amigo. Twenty-four PCR-based landmark unique gene (PLUG) markers from the long arm of wheat homoeologous group 3^17^ were selected and synthesized (Sigma-Aldrich, Inc., St. Louis, MO, USA). PCR were performed according to the procedure in Li et al.^31^. PCR products were digested with *Taq*I (at 65 °C) or *Hae*III (at 37 °C) (Genesearch Pty Ltd, Arundel, QLD, Australia) for improving levels of polymorphism, and separated in 1.5% agarose gels. PLUG markers present in Agent, Janz, Sunco and Amigo but absent in Aroona, CS, AvS and Schomburgk were identified as chromosome 3AgL-specific. The physical locations of PLUG markers were searched in the reference genome sequence of CS (IWGSC RefSeq v.2.1; https://urgi.versailles.infra.fr/blast/).

### Development of NBS-LRR-related molecular markers identifying the smallest alien segment

Firstly, the chromosome 3AgL bin FL 0.85–1.00 containing *Lr24* and *Sr24* were mapped to chromosomes 3E, 3J, 3J^s^ and 3St to determine their physical positions in the reference genome sequences of *Th. elongatum*^32^ (EE, 2*n* = 2*x* = 14) and *Th. intermedium* (JJJ^s^J^s^StSt, 2*n* = 6*x* = 42; https://phytozome-next.jgi.doe.gov/), respectively*.* Depending on the physical positions, we retrieved and extracted all annotated genes and protein sequences in the targeted intervals in chromosomes 3E, 3J, 3J^s^ and 3St from the WheatOmics 1.0 (*Th. elongatum* v1.0; http://202.194.139.32/jbrowse.html) and Phytozome 13 (*Th. intermedium* v3.1; https://phytozome-next.jgi.doe.gov/jbrowse/index.html) databases in combination with the TBtools-II (Toolbox for Biologists) v1.120 software^33^. The Hidden Markov Model (HMM) (https://www.ebi.ac.uk/Tools/hmmer/) profile of the NB-ARC domain (Pfam accession number: PF00931; http://pfam-legacy.xfam.org/) was used as query to perform HMMsearch using the HMMER-3.0 package against the protein sequences of *Th. elongatum* and *Th. intermedium* with an expected value (e-value) threshold of < 1e−30 using a local server MobaXterm. The results were further confirmed using the NCBI Conserved Domain Database (CDD) tool (e-value = 1e−2) (https://www.ncbi.nlm.nih.gov/Structure/cdd/). NBS-LRR candidate genes without a conserved NBS domain were manually removed. Finally, we used an online primer design website Primer-BLAST (https://www.ncbi.nlm.nih.gov/tools/primer-blast/) to design and develop chromosome 3AgL-specific primers from the CDS sequence of the candidate genes.

### Ethical approval

We comply with relevant guidelines and legislation regarding the sample collection and use in the present study. All materials in the present study are not endangered.

### Supplementary Information

Supplementary Legends.

Supplementary Figure 1.

Supplementary Figure 2.

Supplementary Table S1.

## Data Availability

All data generated or analysed during this study are included in this published article and its supplementary information files.
