# Supplementary material for: Cytological and molecular characterization of wheat lines carrying leaf rust and stem rust resistance genes Lr24 and Sr24
Source: Sci Rep. 2024 Jun 4;14:12816. doi: 10.1038/s41598-024-63835-w (PMC11150516; doi:10.1038/s41598-024-63835-w)
Supplement: Supplementary file 1 — Supplementary Legends. [file 41598_2024_63835_MOESM1_ESM.docx]

**Cytological characterization and molecular marker development for wheat-*Thinopyrum ponticum* translocations carrying leaf rust and stem rust resistance genes *Lr24* and *Sr24***

Jianbo Li^1^, Haixia Guan^1^, Yuqi Wang^1,2^, Chongmei Dong^1^, Richard Trethowan^1^, Robert A. McIntosh^1,*^, Peng Zhang^1,*^

^1^ Plant Breeding Institute, School of Life and Environment Sciences, The University of Sydney, 107 Cobbitty Road, Cobbitty, New South Wales 2570, Australia

^2^ Triticeae Research Institute, Sichuan Agricultural University, Chengdu 611130, Sichuan, China

*Corresponding author

Peng Zhang

E-mail: [peng.zhang@sydney.edu.au](mailto:peng.zhang@sydney.edu.au)

Robert A. McIntosh

E-mail: [robert.mcintosh@sydney.edu.au](mailto:robert.mcintosh@sydney.edu.au)

**Supplementary Table 1** NBS-LRR-related genes in the targeted intervals in chromosomes 3E, 3J, 3J^s^ and 3St corresponding to the *Lr24/Sr24* region.

**Supplementary Figure 1.** Uncropped gel images for Fig. 3.

**Supplementary Figure 2.** Uncropped gel images for Fig. 4.
