# Supplementary material for: Cytological and molecular characterization of wheat lines carrying leaf rust and stem rust resistance genes Lr24 and Sr24
Source: Sci Rep. 2024 Jun 4;14:12816. doi: 10.1038/s41598-024-63835-w (PMC11150516; doi:10.1038/s41598-024-63835-w)
Supplement: Supplementary file 2 — Supplementary Figure 1. [file 41598_2024_63835_MOESM2_ESM.pptx]

## Slide 1
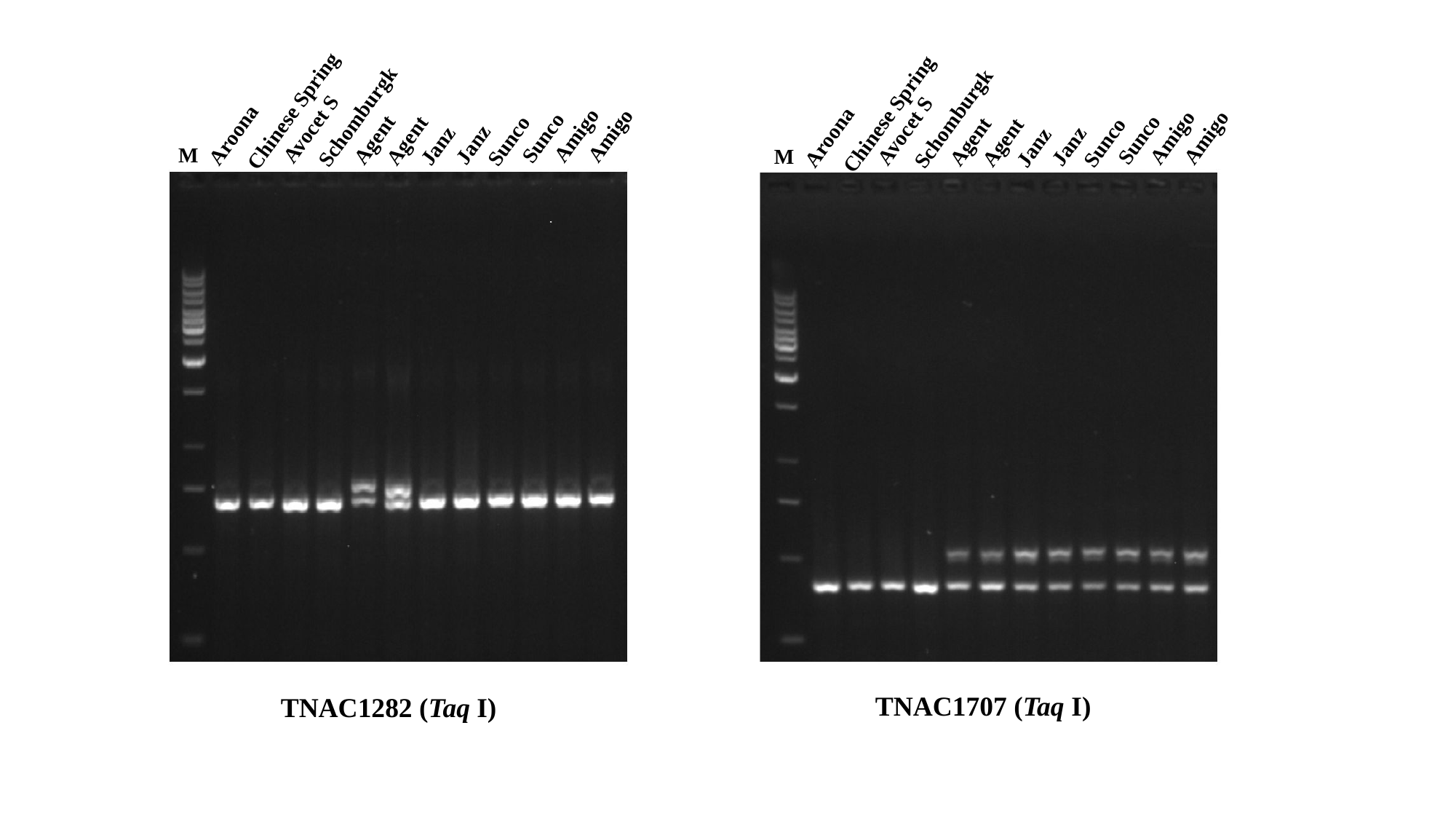

Chinese Spring
Chinese Spring
Schomburgk
Schomburgk
Avocet S
Avocet S
Aroona
Amigo
Amigo
Aroona
Amigo
Amigo
Sunco
Sunco
Agent
Agent
Sunco
Agent
Agent
Sunco
Janz
Janz
Janz
Janz
M
M
TNAC1707 (Taq I)
TNAC1282 (Taq I)
