# Supplementary material for: Cytological and molecular characterization of wheat lines carrying leaf rust and stem rust resistance genes Lr24 and Sr24
Source: Sci Rep. 2024 Jun 4;14:12816. doi: 10.1038/s41598-024-63835-w (PMC11150516; doi:10.1038/s41598-024-63835-w)
Supplement: Supplementary file 3 — Supplementary Figure 2. [file 41598_2024_63835_MOESM3_ESM.pptx]

## Slide 1
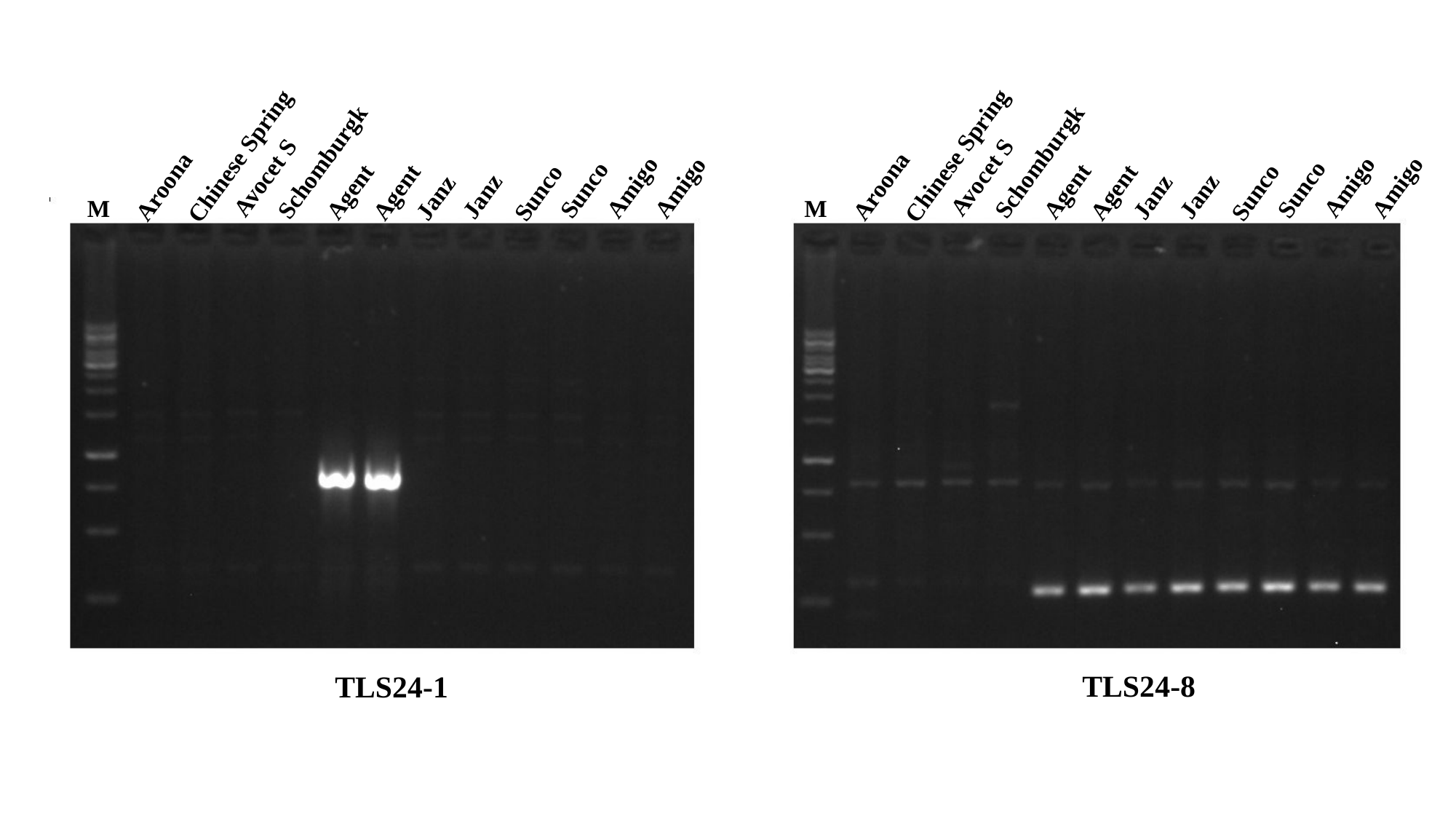

Chinese Spring
Chinese Spring
Schomburgk
Schomburgk
Avocet S
Avocet S
Aroona
Aroona
Amigo
Amigo
Amigo
Amigo
Sunco
Sunco
Agent
Agent
Agent
Agent
Sunco
Sunco
Janz
Janz
Janz
Janz
M
M
TLS24-8
TLS24-1

## Slide 2
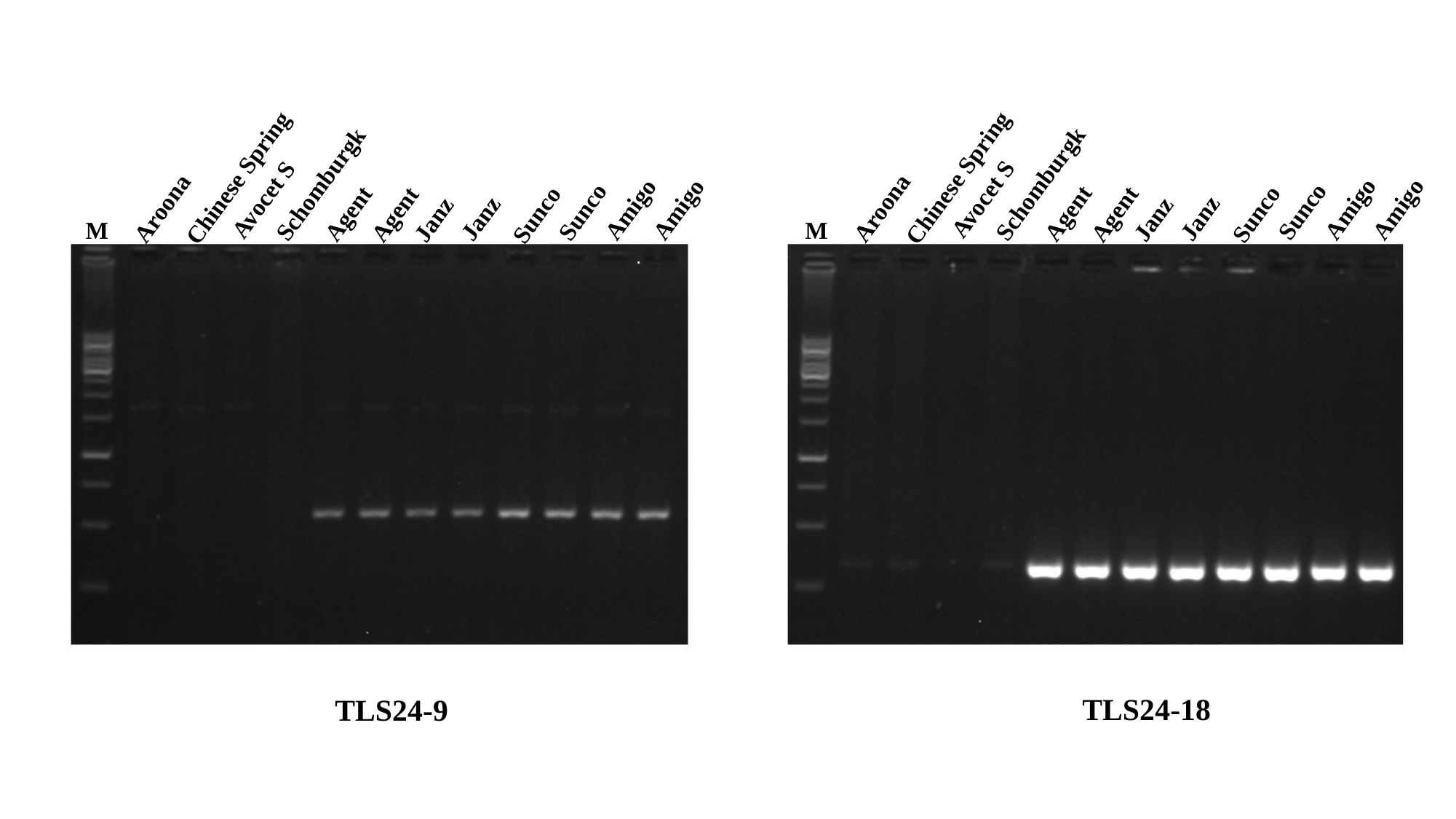

Chinese Spring
Chinese Spring
Schomburgk
Schomburgk
Avocet S
Avocet S
Aroona
Aroona
Amigo
Amigo
Amigo
Amigo
Sunco
Sunco
Agent
Agent
Agent
Agent
Sunco
Sunco
Janz
Janz
Janz
Janz
M
M
TLS24-18
TLS24-9
